# Ohmic Heating Application on Pork Burger Processing

**DOI:** 10.1111/1750-3841.71391

**Published:** 2026-08-22

**Authors:** Celso F. Balthazar, Jonas T. Guimaraes, Rodrigo N. Cavalcanti, Andrea G. Mascarenhas, Thais R. C. Pereira, Renata S. L. Raices, Monica Q. Freitas, Erick A. Esmerino, Adriano G. Cruz, Sergio B. Mano, Eliane T. Mársico

**Affiliations:** ^1^ Department of Veterinary Hygiene and Technological Processing of Animal Products, Veterinary School Federal Fluminense University (UFF) Niterói Brazil; ^2^ Department of Food Technology Federal Institute of Rio de Janeiro (IFRJ) Rio de Janeiro Brazil

**Keywords:** bioactivity, energy efficiency, microbial inactivation, rheological characteristics, sustainability, vitamin B12

## Abstract

This study evaluates ohmic heating (OH) at varying electric field strengths (OH10: 5 V/cm, OH12.5: 6.3 V/cm, OH15: 7.5 V/cm) compared to conventional grilling (CONV) in pork burger processing, focusing on microbial inactivation, physicochemical and rheological properties, and in vitro bioactivity. CONV required 7 min to reach 75°C (heating rate: 11.15°C/min). OH required substantially longer times: 17 min (OH15), 36 min (OH12.5), and 82 min (OH10)—a trade‐off between energy efficiency and throughput that must be considered for industrial application. Regarding energy performance, CONV consumed 996 ± 6 kJ/kg, while OH15 consumed 594 ± 17 kJ/kg—a 40% reduction that was statistically significant (*P* = 0.0097). OH15 significantly reduced total aerobic mesophilic bacteria (1.7 log CFU/mL) compared to CONV (3.6 log CFU/mL; *P* < 0.05), with *Enterobacteriaceae* undetectable in all heated samples. OH maintained macronutrient profiles but reduced water‐holding capacity and increased firmness (76–96 kPa vs. 36 kPa for CONV), indicating textural changes. OH enhanced in vitro bioactivity (DPPH inhibition: 0.51% for OH15 vs. 0.29% for CONV; ACE inhibition: 0.40% vs. 0.15%) and vitamin B12 retention (0.14 vs. 0.11 µg/100 g; *P* < 0.05). However, these bioactivity values represent very low absolute levels and do not support claims that OH‐processed burgers are a source of antioxidant, antihypertensive, or antidiabetic activity. Despite its energy efficiency and microbial inactivation benefits, OH's longer processing time—particularly at lower voltages—presents a challenge for industrial scale‐up. Future work should focus on optimizing electric field strength to balance energy savings, processing time, and product quality.

**Practical Applications:**

This research demonstrates that ohmic heating (OH) offers a more energy‐efficient alternative to conventional grilling for pork burger processing, achieving up to 40% energy savings. By applying electrical current directly through the food, OH provides uniform volumetric heating that effectively inactivates spoilage microorganisms. However, this energy advantage comes with a trade‐off in processing time. Nevertheless, a mathematical projection of OH at equivalent CONV electrical power showed that applying the same peak power (635 W) to OH in a realistic scenario for industrial equipment, it would yield a projected processing time of 3 min (57% faster than CONV's 7 min) while delivering the same total energy (0.03 kWh). This projection reinforces that higher‐power OH systems could overcome current processing time limitations. Claims of global sustainability benefits would require supporting life cycle assessment (LCA) data, which was beyond the present scope. Future pilot‐scale studies are needed to evaluate real‐world throughput, assess consumer acceptance through sensory analysis, and incorporate LCA to quantify environmental trade‐offs. These findings provide a data‐driven foundation for the food industry to weigh the benefits and limitations of OH processing.

## 1 Introduction

The global pork market is a cornerstone of the food system, producing 124.5 million tons in 2023. China leads this output, contributing 57.94 million tons (46.5%), while the United States is the third‐largest producer and a major exporter, underpinning both economic and food security frameworks (USDA FAS 2024). As the most consumed meat worldwide, pork is prized for its affordability, cultural significance, and nutritional value, with demand in many countries projected to grow 8% by 2033 (USDA FAS 2024). Its environmental footprint is also comparatively lower, being 40% more affordable and having a 35% smaller greenhouse gas impact per protein portion than beef (Coelho et al. 2023).

Nutritionally, pork provides high‐quality protein and a significant source of vitamin B12, essential for neurological and cardiovascular health (Gharibzahedi et al. 2023). It also contains bioactive peptides with demonstrated antioxidant, antihypertensive, and antidiabetic properties (Leong and Chang 2024). However, meat processing faces significant hurdles. Microbial safety risks from emerging pathogens are a major concern. These risks are exacerbated by consumer demand for minimally processed and additive‐free foods. Furthermore, conventional thermal methods impose a substantial environmental burden (Michel et al. 2024). Because the food industry's energy inefficiency contributes significantly to global emissions, with forecasts predicting 1.4 trillion metric tons of greenhouse gases by 2100, highlighting an urgent need for sustainable alternatives (Javed et al. 2025).

Ohmic heating (OH) has emerged as a promising sustainable technology to address these challenges. It offers exceptional energy efficiency, with conversion rates often exceeding 90%, directly supporting Sustainable Development Goals for clean energy and climate action (Javed et al. 2025; Gavahian et al. 2025). Studies demonstrated that OH can reduce processing time (Ferreira et al. 2021), lowering energy consumption in food processing and improving energy efficiency by nearly 50% in pasteurization compared to conventional methods (Kriem et al. 2023; Lu et al. 2025). By combining thermal and electroporation effects, OH achieves effective microbial inactivation at lower temperatures, better preserving food quality, extending shelf‐life, and aiding waste valorization (Sain et al. 2024).

However, critical perspectives on ohmic heating (OH) emphasize that the process is not purely thermal but involves complex electrochemical phenomena at the electrode‐food interface that pose significant technical and safety challenges (Sarkis et al. 2013). Literature identifies “undesirable electrochemical reactions” (faradaic) as a primary concern, as they can lead to partial electrolysis of the solution, nutrient degradation and electrode fouling (Ferreira et al. 2021). A major barrier to large‐scale industrial application is electrode corrosion and subsequent metal migration; studies show that stainless steel and even titanium electrodes can release detectable levels of iron, nickel, chromium or titanium into the food matrix, particularly under acidic conditions and low electrical frequencies (e.g., 50–60 Hz, Ferreira et al. 2021).

Despite these challenges, titanium remains a choice for electrode material due to its biocompatibility and non‐toxicity; even trace migration does not compromise the fundamental safety or integrity of the food product (Kim et al. 2022). In comparative studies of ohmic heating, titanium has proven to be a significantly more stable material than stainless steel, with metal release levels demonstrated to be far lower under similar processing conditions (Ferreira et al. 2021).

While OH research has largely focused on liquid foods (Astrain‐Redin et al. 2024), its application to solid matrices like meat remains underexplored (Balthazar et al. 2024). Therefore, this study aims to evaluate the efficiency of OH in pork burger processing, investigating its impact on microbial spoilage inactivation, physicochemical and rheological characteristics, and retention of bioactive compounds.

## 2 Material and Methods

### 2.1 Sample Preparation

Pork burgers were prepared in three independent weekly batches (experimental triplicate). Burger patties were prepared (Table 1) with pork shank and seasonings (salt, black pepper, nutmeg, cumin, oregano, garlic), purchased from a local market in Niterói, Rio de Janeiro, Brazil. Pork shank was manually cut into cubes, then minced and mixed with seasonings in a cutter. From the resulting emulsion, 180 g portions were weighed, shaped into patties (*Ø* 11.2 cm, 2 cm height) using a burger press, and frozen overnight at −18°C. Before heat processing, patties were thawed at room temperature (22°C ± 1) for 2 h.

**TABLE 1:** Ingredients amount (%) per each pork burger sample produced.

Ingredient	Amount (%)
Pork shank (w/w)	96.00
Salt powder (w/w)	2.00
Black pepper powder (w/w)	0.25
Nutmeg powder (w/w)	0.25
Cumin powder (w/w)	0.50
Oregano flakes(w/w)	0.50
Garlic flakes (w/w)	0.50

Samples were divided into five treatment groups: CTRL (unprocessed raw burger); CONV (processed on a conventional electric grill); OH10, OH12.5 and OH15 (processed by ohmic heating at different voltage settings (see below).

### 2.2 Heat Processing Methods

Conventional grilling (CONV) was performed using a 127 V electric grill (Fast Grill S‐12, Mondial, Barueri, Brazil).

Ohmic heating (OH) was conducted using a custom unit consisting of a voltage variator (TDGC2‐2kVA, JNG Electrical Materials, São Paulo, Brazil) with adjustable output (0–220 V). Connected to a 220 V, 60 Hz AC sinusoidal power supply, the unit delivered current through titanium electrodes (200 × 250 mm, 1.6 mm thickness) placed 2 cm apart. Burger patties placed between the electrodes generated electric field strengths of 5, 6.3, and 7.5 V/cm, corresponding to applied voltages of 10 V (OH10), 12.5 V (OH12.5), and 15 V (OH15).

**FIGURE 1:**
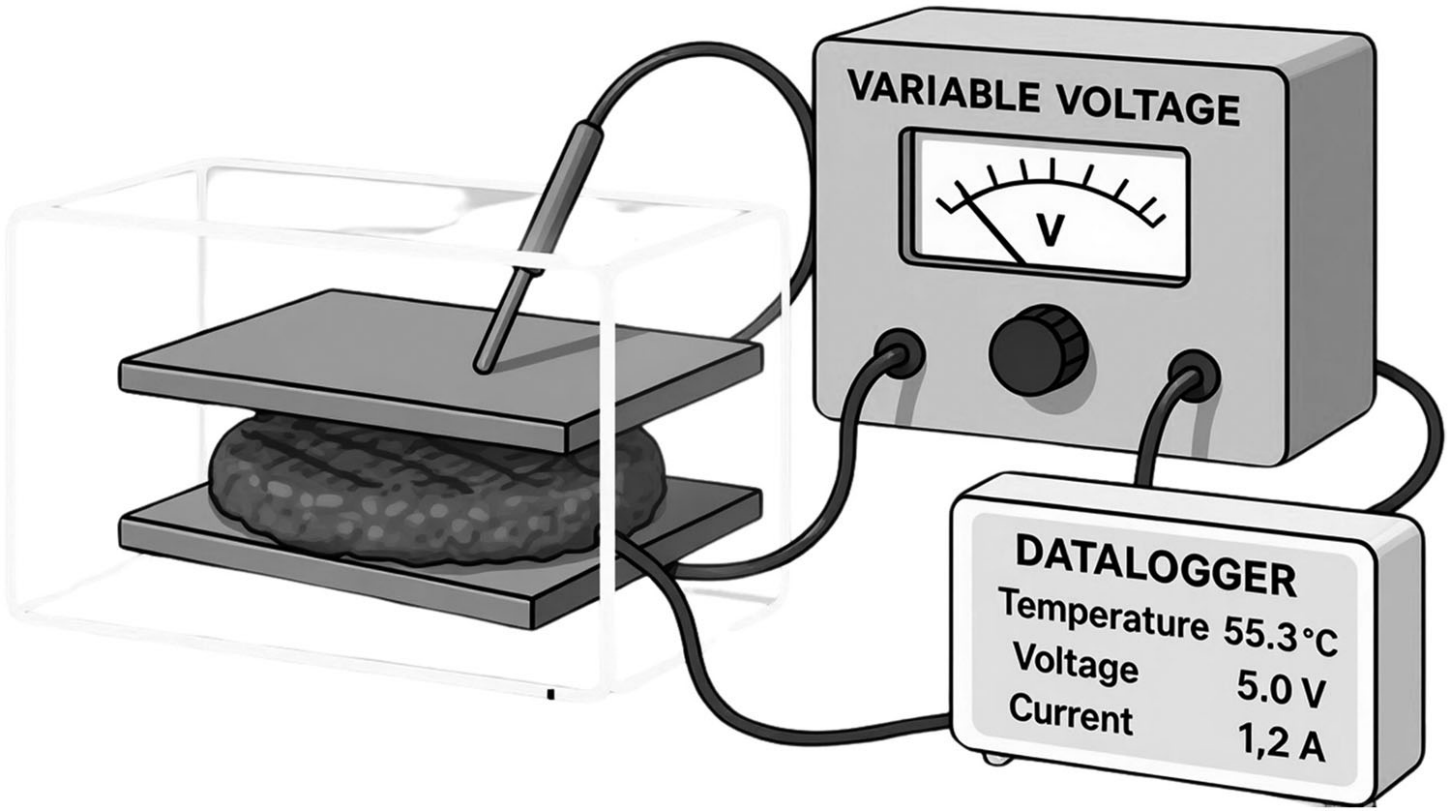
Scheme of ohmic processing system.

Those voltages were chosen based on preliminary screening over a wider range. Voltages below 10 V produced processing times exceeding practical limits for industrial or home use (data not shown). Voltages above 15 V generated currents that risked damaging the ohmic heating unit, given the specific electrical resistance of the burger patties. Due to that equipment limitation, 15 V was set as the upper operational limit, and 10 V as the lower practical limit. The burger patty between electrodes was maintained in a polycarbonate container (240 × 260 × 105 mm) during OH processing (Figure 1) to avoid heat dissipation.

For both methods, patties were heated until the internal temperature reached 75°C and maintained at that temperature for 15 s. Voltage, current, and temperature were recorded in a digital datalogger (RTR500B Series, T&D, Japan) for subsequent electrical parameter analyses using Microsoft Excel.

### 2.3 Electrical Parameters

Electrical parameters were calculated following Balthazar et al. (2022) equations presented in Table 2. For CONV, energy consumption and specific energy consumption were calculated using the same formulas, based on measured voltage (127 V) and current (approximately 5 A). The grill's thermostatic control caused intermittent current draw (zero readings when the heating element cycled off). Therefore, only intervals with non‐zero current were included in the calculation.

**TABLE 2:** Equations of ohmic heating electrical parameters and energy consumption for burger heating processes.

Parameter	Equation	Symbol
Electric field strength	*E* = *V*/*d*	V/cm
Electrical conductivity	*σ* = *IL*/(*AV*)	S/m
Heat generation	HG = *σ×|ΔE|^2^*	mW/m
Volumetric heat generation	*VHG* = *σ×|ΔE|^2^vd*	µW/cm^3^
Electrical power	*P = V x I*	W
Energy consumption	*C* = ∑(*V I t*)	kWh
Specific energy consumption	SEC *= C* x 3600/*m*	kJ/kg

*Note: V* = voltage (V), *d* = electrode distance (cm), *I* = current (A), *L* = distance between electrodes (m), *A* = electrode surface area (m^2^), *vd* = burger volume (m^3^), and *t* = time interval (h or s), *m* = initial burger mass (kg), 3600 = conversion factor from kWh to kJ.

Heating rate (°C/min) was obtained from temperature–time curves fitted to linear regression (*T *=* at + b*), where *a* is the heating rate.

### 2.4 Microbiological Analysis

Burger samples (25 g) were aseptically weighed and transferred into sterile stomacher bags. Each sample was homogenized with 225 mL of sterile 0.9% NaCl (w/v) solution using a stomacher (Stomacher 400, Seward, UK) at 230 rpm for 2 min, yielding a 10^−1^ dilution. Subsequent tenfold serial dilutions were prepared by transferring 1 mL of the homogenate into 9 mL of sterile 0.9% NaCl (w/v) solution and vortexing for 30 s, ensuring that the 1 mL transferred to the next tube is truly representative of the whole mixture.

Total aerobic mesophilic bacteria (TAMB) were quantified using the spread plate method. Aliquots (0.1 mL) of appropriate dilutions were spread onto Plate Count Agar (PCA, CM0325, Oxoid, Hampshire, England) and incubated at 32 ± 0.5°C for 24–48 h.

*Enterobacteriaceae* counts were determined using the pour plate method. Aliquots (1 mL) of appropriate dilutions were dispensed into sterile Petri dishes, mixed with Violet Red Bile Glucose Agar (VRBG, CM0485, Oxoid, Hampshire, England), and allowed to solidify. An overlay of the same medium was added, and plates were incubated at 35 ± 0.5°C for 24–48 h.

Lactic acid bacteria (LAB) were counted using the pour plate method with MRS agar (CM0359, Oxoid, Hampshire, England). After solidification, an MRS agar overlay was added, and plates were incubated at 35 ± 0.5°C for 48–72 h (APHA 2015).

All results were expressed as colony‐forming units per gram of sample (CFU/g). Each dilution was plated in duplicate and analyses were performed in triplicate across three independent batches.

### 2.5 Physicochemical Analyses

Cooking loss was determined by weighing each burger patty before and after heat processing. Weight loss percentage was calculated as:

(1) $$Cookingloss\left(\%\right)=\left(W_{before}-W_{after}/W_{before}\right)\times 100$$

where *W*_before_ and *W*_after_ are the sample weights before and after heating, respectively.

Water‐holding capacity (WHC) was determined according to the method described by Ángel‐Rendón et al. (2020). Briely, 1.500 ± 0.100 g burger sample was wrapped in a pre‐weighed No. 3 filter paper and further wrapped in a No. 50 filter paper. The wrapped samples were centrifuged at 9000 × *g* for 20 min at 4°C using a Sorvall ST16R, Thermo Scientific (São Paulo, Brazil). Thereafter, No. 3 filter paper containing the sample was weighed, and WHC was calculated using the following equation:

(2) $$\mathrm{WHC}\;\left(\%\right)=\frac{\left(W\mathrm{sp}-Wp\right)}{Ws\;\times \;\mathrm{MC}}\times 100$$

where, *W*_sp_ is the final weight of the sample plus No. 3 filter paper after centrifugation (g); *W*_p_ is the initial weight of the No. 3 filter paper (g); *W*_s_ is the initial sample weight (g); and MC = moisture content of the sample on a wet basis (decimal).

The proximate composition analysis was conducted following standard protocols (AOAC 2005). Moisture content was quantified gravimetrically according to AOAC method 930.15. Samples were dried in an oven at 105 ± 1°C until constant mass was achieved, and measurements were recorded to 0.1 mg precision. Moisture content was calculated as the percentage of weight lost during drying. Total protein content was determined using the micro‐Kjeldahl method (AOAC 984.13). Organic nitrogen was converted to ammonium sulfate via acid digestion, followed by distillation and titration. The nitrogen content was subsequently converted to crude protein using the standard conversion factor of 6.25 for animal proteins. Lipid content was determined by Soxhlet extraction (AOAC 920.39). Samples were completely dried prior to extraction to prevent hydrolysis interference. Petroleum ether (40°C–60°C boiling range) was used as the organic solvent. Total lipid content was calculated from the weight difference pre‐ and post‐extraction. Ash content was determined according to AOAC method 942.05. Samples were incinerated in a muffle furnace at 550 ± 5°C for 6 h. Crucibles were cooled in a desiccator before gravimetric measurement. Ash content was calculated as the percentage of remaining inorganic residue. Dietary fiber content was not directly measured in the burger samples. Instead, it was estimated based on the declared nutritional information of the seasoning ingredients (salt, black pepper, nutmeg, cumin, oregano, and garlic) provided by the manufacturer, combined with the fiber content of pork shank (negligible). This approach was necessary because the seasoning mixture contributed the total of dietary fiber to the final product. Carbohydrate content was calculated by difference of moisture, protein, lipid, ash, and fiber).

The pH was measured with a digital potentiometer (PG1800, Chapter Lab, Brazil). Water activity (*a*_w_) was determined using a LabSwift‐aw device (Novasina, Switzerland).

Lipid oxidation (thiobarbituric acid reactive substances—TBARS) followed Buege and Aust (1978) method. Briefly 5 g sample was homogenized with 22.5 mL of 11% trichloroacetic acid, centrifuged (11,000 × *g*, 4°C, 15 min). Supernatant (1 mL) was mixed with 1 mL of 20 mM thiobarbituric acid, incubated at 25°C for 20 h, and absorbance read at 532 nm. Results expressed as mg malondialdehyde (MDA)/kg.

Instrumental color was measured using a CM‐600D spectrophotometer (Konica Minolta, Japan; d/8° geometry, 10° observer, D65 illuminant). Five random readings were taken per sample (22 ± 1°C, <500 lux ambient light) and parameters L* (lightness), a* (red–green), b* (yellow–blue) recorded. From those, color metrics (hue angle—*h°*, chroma—C*, and total color difference—∆E*) were calculated according to Guerrero et al. (2025):

(3) $$C*=\sqrt{\left(a{*}^{2}+b{*}^{2}\right)}$$

(4) $$h^{\circ }=arctan\left(b*/a*\right)$$

(5) $$\Delta E*=\surd \Delta \Delta {*}^{2}+\Delta \alpha {*}^{2}+\Delta b{*}^{2}$$

### 2.6 Rheological Analysis

Measurements were performed with a TA‐XT21 texture analyzer (Stable Micro Systems, UK; 50 kg load cell, 35 mm aluminum plate). Cylindrical samples (20 mm diameter × 20 mm height) were refrigerated at 4°C around 90 min, as five replicates per sample.

Uniaxial compression to 20% of initial height (crosshead speed 1 mm/s) was acquired, and true stress (*σ*_t_, Equation 6) and true strain (ε_t_, Equation 7) were calculated as described by Santos et al. (2015):

(6) $$\sigma_{t}=\frac{F\left(t\right)}{A\left(t\right)}=\frac{F\left(t\right)L\left(t\right)}{A_{0}L_{0}}\;$$

(7) $$\varepsilon_{t}=\left|\ln\left(\frac{L\left(t\right)}{L_{0}}\right)\right|=\left|\ln\left(\frac{L_{0}-\Delta L}{L_{0}}\right)\right|\;$$

where 𝐹(𝑡) is the applied force at time, 𝐴(𝑡) is the cross‐sectional area at time, 𝐴_0_ is the initial cross‐sectional area, 𝐿(𝑡) is the sample length at time, 𝐿_0_ is the initial sample length, and 𝛥𝐿 is deformation.

Stress–strain data were fitted to a fifth‐order polynomial through the origin (Equation 8), as described by Santos et al. (2015). The modulus of deformability 𝐸_𝐷_ was determined from the initial linear region of the stress‐strain curve (Equation 9). Peak strain (𝜀_20_) was identified at the peak stress point where the slope becomes zero (𝑑𝜎_𝑡_⁄𝑑𝜀_𝑡_ = 0), and the corresponding stress value was defined as the peak stress (𝜎_20_). The work of 20% compression (𝑊_20_) was calculated as the area under the stress‐strain curve up to the peak point (Equation 10):

(8) $$\sigma_{t}=\sum_{i=1}^{5}a_{i}\varepsilon_{t}^{i}\;$$

(9) $$E_{D}={\left[\frac{d\sigma_{t}}{d\varepsilon_{t}}\right]}_{\varepsilon_{t}\to 0}\;$$

(10) $$W_{20}=\int_{0}^{\varepsilon_{20}}\sigma_{t}d\varepsilon_{t}\;$$

Creep tests were conducted by applying a constant force of 0.89 N to the samples for 180 s. Subsequently, the force was removed, and recovery was monitored for an additional 180 s. Force–displacement data were converted to stress–strain values. Compliance, defined as the ratio of strain at time t to the applied stress ((𝑡) = (𝑡) ⁄ 𝜎0), was calculated for each time point. The compliance data were fitted to a four‐component Burger model using nonlinear regression (Equation 11).

(11) $$J\left(t\right)=J_{0}+J_{1}\left(1-e^{-t/\tau }\right)+\frac{t}{\eta_{N}}$$

where *J(t)* is the compliance over time, *J*_0_ is the instantaneous elastic compliance associated with the Maxwell portion, *J*_1_ is the viscoelastic compliance related to the Kelvin–Voigt element, *τ* is the retardation time related to the Kelvin–Voigt element, *η_N_* is the Newtonian viscosity associated with the Maxwell portion.

### 2.7 Volatile Organic Compounds (VOCs) by Electronic Nose

VOCs were analyzed using a Heracles II electronic nose (Alpha M.O.S., France) with dual columns (MXT‐5, MXT‐1701), dual FIDs, and HS100 autosampler. Samples (4 g) in 20 mL vials were stored at –18°C, then incubated at 80°C for 15 min. Headspace (5 mL) was injected at 200°C. Identification used C6–C16 alkanes and the AroChemBase library (Kovat's index). Oven program starting at 50°C, ramping to 250°C. Discriminant Function Analysis (DFA) of the VOC data is described in Section 2.9.

### 2.8 Biological Functionality

To evaluate functional properties, samples were first subjected to an extraction process using acidified (HCl) water (pH 4.6), which was stirred for 5 min to precipitate casein. The mixture was then centrifuged at 10,000 × *g* for 15 min at 4°C. The resulting supernatants were isolated through filtration using a 0.45‐µm syringe filter and stored at −20°C until needed for bioactive compound analyses, according to Ayyash et al. (2018).

The antioxidant activity of the extracts was measured using the 2,2‐diphenyl‐1‐picrylhydrazyl (DPPH) radical scavenging method. The scavenging activity, expressed as a percentage of inhibition, was calculated by comparing the sample's absorbance at 517 nm against a control absorbance recorded at the same wavelength (Equation 12).

(12) $$\mathrm{DPPHI}\;\left(\%\right)=\left(1-\frac{\mathrm{Abs}\;\mathrm{sample}}{\mathrm{Abs}\;\mathrm{blank}}\right)\;\times 100$$

The angiotensin I‐converting enzyme inhibitor (ACEI), antihypertensive activity, was determined using a spectrophotometric assay (absorbance at 228 nm). The percentage of ACEI activity was calculated using the following equation:

(13) $$\mathrm{ACEI}\;\left(\%\right)=\left[\frac{\left(B-A\right)}{\left(B-C\right)}\right]\times 100$$

where *A* represented the absorbance of the solution containing the sample (with inhibitor) and the specific ACEI components, *B* represented the absorbance of the solution without the inhibitor component, and *C* is the absorbance of the solution with no inhibitor present.

The antidiabetic potential was assessed through the inhibition of digestive enzymes, specifically α‐amylase and α‐glucosidase. For the α‐amylase assay, activity was determined by measuring the absorbance at 540 nm. For α‐glucosidase, activity was quantified by measuring the release of p‐nitrophenol at 400 nm. Solutions prepared without the sample extract or without the substrate were utilized as controls and blanks, respectively. The final percentage of enzyme inhibition was calculated as:

(14) $$\begin{array}{ccc} & & \mathrm{Enzime}\;\mathrm{inhibition}\;\left(\%\right)\\ & & =[1-\left.\left(\;\frac{\left(\mathrm{Abs}\;\mathrm{sample}\;-\;\mathrm{Abs}\;\mathrm{blank}\right)}{\mathrm{Abs}\;\mathrm{control}}\right.\right)]\;\times 100 \end{array}$$

Vitamin B12 (cobalamin) was quantified by HPLC (Czerwonka et al. 2014) using a Waters Alliance 2690 system with a PDA detector and C18 column (4.6 × 250 mm, 5 µm). Samples underwent acid hydrolysis (0.1 M HCl, 121°C, 30 min), were subjected to enzymatic hydrolysis (takadiastase, 37°C, 18 h), and protein precipitation by trichloroacetic acid (TCA) in a water‐boiling system for 10 min. Mobile phase consisted of 0.1% trifluoroacetic acid (TFA) in water/acetonitrile (0.3–0.5 mL/min). Detection was performed using a UV/vis photodiode array detector at 361 nm, for a total run time of 25 min.

### 2.9 Statistical Analysis

The experiment was performed in triplicate across three different weeks (batches), and analyses were carried out at least in triplicate within each batch. Data were evaluated in XLSTAT version 2019.2 (Adinsoft, Paris, France) using linear mixed models (LMMs) where appropriate, with approximate *F*‐ratio tests for fixed effects and significance at *P* ≤ 0.05. Predicted means ± standard errors were generated, and pairwise comparisons used Fisher's least significant difference (LSD) at the 5% level. Non‐significant terms were removed via stepwise backward elimination.

Models for electrical parameters, energy consumption, heating rate, microbiological analyses, physical and chemical analyses, rheological analyses, and biological functionality included heat processing treatment (CTRL, CONV, OH10, OH12.5, OH15) as a fixed effect and batch as a random effect. For rheological analyses, replicate (nested within batch) was also included as a random effect. Interactions between treatment and repeated measures (e.g., time for creep tests) were tested and retained if significant.

DFA model was constructed using Alpha MOS electronic nose software to classify burger samples based on their VOC profiles. Sensors with relative standard deviation below 50% were selected for model building. The model was trained using nine replicates of raw pork shank (MEAT), unprocessed raw burger (CTRL), and conventionally grilled burger (CONV) as anchor groups. The recognition rate was calculated from the training set using cross‐validation, confirming internal consistency and reliable differentiation among the anchor groups.

Six replicates of each ohmic‐heated sample (OH10, OH12.5, OH15) were then projected into the anchor‐defined discriminant space to assess their similarity to the anchor groups. Because the OH samples were not included in model training, their classification represented a projection rather than a cross‐validated prediction. A leave‐one‐out cross‐validation across all samples (including OH) was not performed, as the primary objective was to evaluate the aromatic similarity of OH samples to conventional grilling, not to develop a predictive classifier.

For the DFA model, Wilks' lambda test was used to assess the significance of each discriminant function. The first two discriminant functions were significant (DF1 and DF2, *P* < 0.05 for both). Additional discriminant functions (DF3, DF4, DF5) were not significant (*P* > 0.05, Wilks' lambda) and are not reported. Batch effects were assessed as a covariate in preliminary models and found to be non‐significant; therefore, they were not included in the final DFA model.

## 3 Results and Discussion

### 3.1 Ohmic Heating and Grilling Heat Processing Evaluation

**FIGURE 2:**
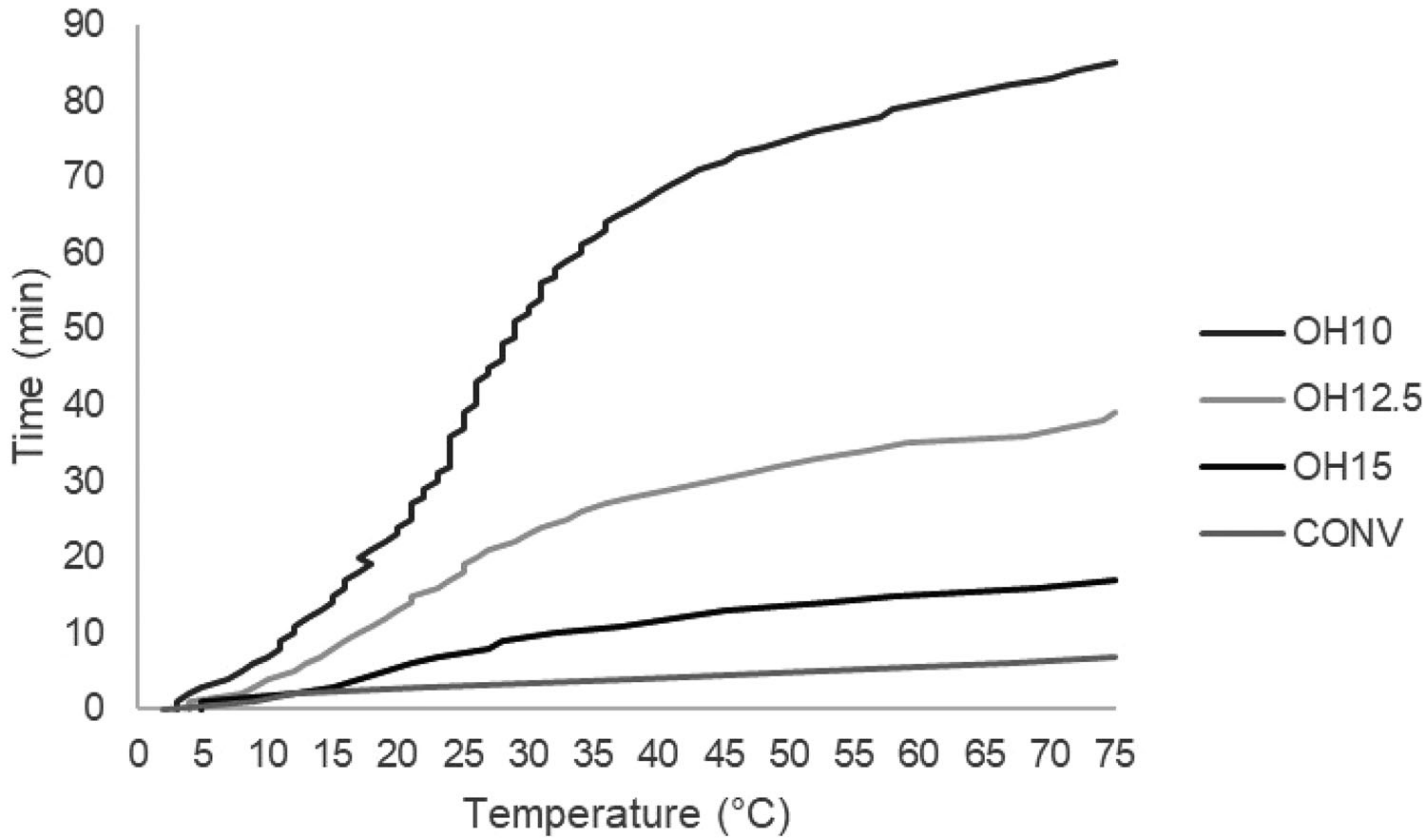
Internal temperature profiles of pork burgers during conventional grilling (CONV) and ohmic heating at 15 V (OH15), 12.5 V (OH12.5), and 10 V (OH10). Error bars were omitted for clarity.

The present study aimed to achieve energy consumption, specific energy consumption, and heating efficiency of pork burgers heated using CONV and OH (OH10, OH12.5, and OH15) to better understand the performance of those processes. Figure 2 shows the mean triplicate internal temperature profiles of pork burgers during heating processes, where the treatments were heated until the internal temperature reached 75°C.

Table 3 summarizes the time and heat parameters for efficient pork burger heat processing. In absolute terms, CONV consumed 0.05 kWh per burger, while OH10, OH12.5, and OH15 consumed 0.04, 0.04, and 0.03 kWh, respectively. Although these absolute differences suggested a trend toward lower energy use with ohmic heating, no significant differences were detected among treatments when considering total energy consumption in kWh (*P* > 0.05).

**TABLE 3:** Heat processing parameters of pork burger by ohmic heating and conventional grilling comprise heat processing time, heating rate (HR), heating generation (HG), volumetric (VHG), and total processing energy consumption.

	Heat processing time (min)	HR (°C/min)	HG (mW/m)	VHG (µW/cm^3^)	*C* (kWh)	SEC (kJ/kg)
CONV	7.00 ± 0.50^d^	11.15 ± 0.04^a^	—	—	0.05 ± 0.01^a^	996 ± 6^a^
OH10	82.00 ± 9.29^a^	0.65 ± 0.07^d^	1.60 ± 0.15^c^	0.32 ± 0.02^c^	0.04 ± 0.01^a^	786 ± 169^ab^
OH12.5	36.00 ± 9.29^b^	1.95 ± 0.42^c^	3.48 ± 0.15^b^	0.68 ± 0.11^b^	0.04 ± 0.02^a^	864 ± 307^ab^
OH15	17.00 ± 2.52^c^	3.95 ± 1.25^b^	5.55 ± 0.15^a^	1.01 ± 0.30^a^	0.03 ± 0.01^a^	594 ± 17^b^

*Note*: Different letters in the same column mean significant difference (*p* < 0.05, Fisher's LSD). HG: heat generation; VHG: volumetric heat generation; *C*: energy consumption; SEC: specific energy consumption; CTRL: unprocessed raw pork burger; CONV: conventional grilled pork burger; OH10: ohmic heated at 5 V/cm pork burger; OH12.5: ohmic heated at 6.3 V/cm pork burger; OH15: ohmic heated at 7.5 V/cm pork burger.

To enable a more equitable comparison of energy efficiency, specific energy consumption (SEC) was performed (Table 3). CONV required 996 kJ/kg, in contrast with OH15 for 594 kJ/kg (*P* < 0.05). This difference could be translated into a 40% energy reduction. OH10 (786 kJ/kg) and OH12.5 (864 kJ/kg) showed intermediate SEC values that did not differ significantly from either CONV or OH15 (*P* > 0.05), likely due to the high within‐treatment variability observed for these conditions. The data suggested that ohmic heating at the highest tested electric field strength offered a meaningful energy efficiency advantage over conventional grilling. This finding aligned with previous studies reporting improved energy efficiency in ohmic heating systems when operated at higher voltages, attributed to more rapid heating and reduced heat losses to the surroundings (Javed et al. 2025). In contrast, the lower voltages applied in OH10 (5 V/cm) and OH12.5 (6.3 V/cm) resulted in prolonged processing times (82 and 36 min, respectively), during which heat dissipation to the environment may have offset some of the theoretical energy savings.

CONV exhibited the fastest heating process time, reaching 75°C at the geometric center in only 7 min, attributed to a high heating rate of 11.15°C/min (P < 0.05), due to the equipment working at an average electrical power of 453 W, as calculated. The heating rate in the OH process increased with rising electric field strength (0.65 for 5 V/cm < 1.95 for 6.3 V/cm < 3.95 for 7.5 V/cm, *P* < 0.05), correspondingly increasing heat generation and volumetric heat generation (*P* < 0.05, Table 3). Moreover, OH processing achieved significantly lower electrical power (OH10 = 26.70 W < OH12.5 = 63.80 W < OH15 = 108.90 W, *P* < 0.05) than CONV, explaining longer processing time, lower heating rate, and similar electrical consumption. This unsatisfactory result was due to OH equipment limitations.

Nevertheless, a mathematical projection of OH at equivalent CONV electrical power would use the mean energy required for OH derived from present data (0.03 kWh) and the projected processing time at 635 W as,

$$t_{\mathrm{projected}}={E_{\mathrm{OH}}}_{\left(\mathrm{actual}\right)}/P_{\mathrm{CONV}}=0.03kWh/0.635\;kW=0.05\;h=3min$$

Conventional grilling operates intermittently due to thermostatic control, drawing 635 W when the heating element is on and 0 W when off, resulting in an average power of 453 W over the total processing time. However, this average does not represent the grill's actual electrical capacity but rather the effect of temperature regulation. Ohmic heating, in contrast, allows continuous power delivery without thermostatic cycling, as heat is generated volumetrically and can be precisely controlled. Therefore, applying the same peak power (635 W) to OH represents a realistic scenario in which OH operates continuously at the power level the grill can only sustain intermittently. Using the lower average power (453 W) would underestimate OH's potential, as it would artificially impose the grill's cycling limitation onto a technology that does not require it.

Consequently, the projection assumes that OH delivers the same total energy required to cook the burger (0.03 kWh) but at a constant power of 635 W, yielding a projected processing time of 3 min, resulting in approximately 57%‐time reduction compared to CONV (7 min).

### 3.2 Microbiology Evaluation of Pork Burgers

Pork burgers were stored at 4°C ± 1 for 24 h before microbiological analysis. In heat‐processed burgers, this procedure allowed for the recovery of sublethally injured cells, thereby avoiding false‐negative results (Shao et al. 2023). As expected, the CTRL sample exhibited the highest TAMB count (5.1 log CFU/g, *P* < 0.05), representing the accompanying microbiota from the meat chain to the market. CONV reduced TAMB significantly to 3.6 log CFU/g (*P* < 0.05), showing the efficiency of the grilling method of processing. Among OH treatments, OH10 and OH12.5 showed similar TAMB counts (2.2 and 2.3 log CFU/g, respectively, *P* > 0.05), while OH15 had the lowest count (1.7 log CFU/g, *P* < 0.05). As well, the present results are consistent with Zell et al. (2010), who reported significant reductions in mesophilic aerobic bacteria in beef meatballs treated with OH at 15.3 V/cm and 75°C, achieving comparable or superior inactivation compared to conventional heating. The enhanced inactivation achieved in OH processing is attributed to the combined thermal and non‐thermal effects of OH, such as electroporation, which increases cell membrane permeability and facilitates microbial death (Singh et al. 2024; Tian et al. 2018).

Consistent with these findings, Maspeke et al. (2024) also reported that increasing electric field strength enhanced microbial inactivation during ohmic heating of meat products. However, it must be acknowledged that the OH treatments in this study also involved substantially longer heating times (17–90 min) compared to CONV (7 min). Therefore, the observed increase in bacterial inactivation could be attributed, at least in part, to prolonged thermal exposure rather than exclusively to electroporation. Thus, a definitive demonstration of the electroporation effect would require a study design in which time‐temperature profiles would be standardized across treatments. This approach would allow isolation of the non‐thermal contribution of the electric field from the thermal effect, highlighting the importance of future studies designed to decouple thermal and non‐thermal effects by matching time‐temperature histories across the OH system.

*Enterobacteriaceae* counts were reduced to undetectable levels (0.0 log CFU/g) in all heated samples (CONV, OH10, OH12.5, and OH15), compared to 3.7 log CFU/g in CTRL (*P* < 0.05). This result was expected, as Enterobacteriaceae are generally susceptible to temperatures above 71°C, which was reached at the geometric center of all cooked burgers (Mladenović et al. 2021).

Lactic acid bacteria (LAB) counts were significantly higher in CTRL (3.7 log CFU/g) compared to all heated samples (*P* < 0.05). Among heated samples, CONV, OH10, and OH15 showed statistically equivalent LAB counts (0.5, 0.2, and 0.5 log CFU/g, respectively; *P* > 0.05), while OH12.5 had undetectable levels (0.0 log CFU/g). No significant differences in LAB counts were observed among any of the heat‐processed samples (*P* > 0.05).

Despite the absence of significant differences, the absolute inactivation pattern across OH treatments was noteworthy. OH12.5 (6.3 V/cm, 36 min) achieved complete LAB inactivation (below detection), whereas OH10 (5 V/cm, 90 min) and OH15 (7.5 V/cm, 17 min) showed residual counts. This pattern suggests that both thermal exposure time and electric field strength contribute to LAB inactivation. OH10, despite its prolonged heating time (90 min), did not achieve the same inactivation level as OH12.5, possibly because the lower electric field strength (5 V/cm) was insufficient to enhance cell membrane permeability. Conversely, OH15 had a higher electric field strength (7.5 V/cm) but the shortest processing time (17 min), which may have limited cumulative thermal exposure—a critical factor for thermotolerant LAB. OH12.5 appears to have benefited from an intermediate combination of sufficient electric field strength (6.3 V/cm) and adequate thermal exposure (36 min), allowing electroporation to act synergistically with thermal effects, resulting in complete inactivation.

### 3.3 Pork Burgers Composition and Physicochemical Properties

The composition and physicochemical properties of pork burgers processed by CONV and OH were evaluated and compared to CTRL. Results for proximate composition and physicochemical parameters are presented in Tables 4 and 5, respectively.

**TABLE 4:** Pork burgers composition.

	Protein (%)	Moisture (%)	Ashes (%)	Fat (%)	Carb (%)	Dietary fiber (%)	
CTRL	28.84 ± 1.51^a^	62.68 ± 2.55^a^	3.17 ± 0.12^a^	4.14 ± 0.98^a^	0.42 ± 0.01^a^		0.76 ± 0.01^a^
CONV	29.21 ± 2.06^a^	61.52 ± 3.56^a^	3.41 ± 0.25^a^	4.80 ± 1.29^a^	0.30 ± 0.22^a^		0.77 ± 0.01^a^
OH10	29.24 ± 0.75^a^	61.51 ± 0.49^a^	3.34 ± 0.26^a^	4.70 ± 0.78^a^	0.43 ± 0.02^a^		0.77 ± 0.03^a^
OH12.5	29.04 ± 0.92^a^	61.54 ± 0.96^a^	3.38 ± 0.36^a^	4.86 ± 1.60^a^	0.42 ± 0.01^a^		0.77 ± 0.01^a^
OH15	29.36 ± 1.29^a^	61.41 ± 0.91^a^	3.32 ± 0.19^a^	4.70 ± 1.34^a^	0.43 ± 0.01^a^		0.77 ± 0.02^a^

*Note*: Different letters in the same column mean significant differences (*p* < 0.05, Fisher's LSD). CTRL: unprocessed raw pork burger; CONV: conventional grilled pork burger; OH10: ohmic heated at 5 V/cm pork burger; OH12.5: ohmic heated at 6.3 V/cm pork burger; OH15: ohmic heated at 7.5 V/cm pork burger.

**TABLE 5:** Pork burgers physicochemical properties.

						Color parameters					
	Cooking loss (%)	pH	WHC (%)	*a*_w_ (%)	Lipox (mg MDA/kg)	L*	a*	b*	C*	h*	∆E*
CTRL	—	5.80 ± 0.17^a^	57.70 ± 4.68^a^	0.94 ± 0.15^a^	0.11 ± 0.01^b^	52.55 ± 5.33^b^	10.08 ± 1,29^a^	16.98 ± 0.62^b^	19.76 ± 1.11^ab^	1.04 ± 0.05^c^	—
CONV	5.81 ± 0.86^a^	5.90 ± 0.17^a^	54.53 ± 3.63^ab^	0.94 ± 0.35^a^	0.12 ± 0.01^b^	53.10 ± 2.42^b^	9.77 ± 0.87^a^	21.54 ± 1.03^a^	23.66 ± 1.11^a^	1.14 ± 0.03^b^	5.97 ± 2.35^a^
OH10	5.68 ± 0.12^a^	5.90 ± 0.10^a^	49.96 ± 3.26^bc^	0.94 ± 0.35^a^	0.16 ± 0.03^a^	60.83 ± 2.49^a^	5.66 ± 1,14^b^	15.85 ± 1.67^b^	16.83 ± 1.93^b^	1.23 ± 0.03^a^	6.60 ± 2.56^a^
OH12.5	6.63 ± 1.00^a^	5.93 ± 0.06^a^	46.06 ± 2.65^c^	0.93 ± 0.36^a^	0.12 ± 0.01^b^	57.14 ± 5.84^ab^	6.71 ± 1.42^b^	19.61 ± 3.32^ab^	20.73 ± 3.59^ab^	1.24 ± 0.02^a^	6.90 ± 2.33^a^
OH15	7.14 ± 1.67^a^	5.83 ± 0.15^a^	57.19 ± 2.27^a^	0.93 ± 0.15^a^	0.12 ± 0.01^b^	58.98 ± 3.66^ab^	6.09 ± 0.73^b^	19.71 ± 3.67^ab^	20.63 ± 3.70a^b^	1.27 ± 0.03^a^	6.64 ± 1.88^a^

*Different letters in the same column means significant difference (*p* < 0.05, Fisher's LSD). WHC: water‐holding capacity; a_w_: water activity; Lipox: lipid oxidation; L*: lightness; a*: red‐green; b*: yellow‐blue; C*: chroma; h*: hue angle; ∆E*: total color difference; CTRL: unprocessed raw pork burger; CONV: conventional grilled pork burger; OH10: ohmic heated at 5 V/cm pork burger; OH12.5: ohmic heated at 6.3 V/cm pork burger; OH15: ohmic heated at 7.5 V/cm pork burger.

As expected, the macronutrient profiles of pork burgers did not differ significantly among treatments (*P* > 0.05, Table 4): protein (28.8%–29.4%), moisture (61.4%–62.7%), ash (3.2%–3.4%), fat (4.1%–4.9%), carbohydrates (0.3%–0.4%), and dietary fiber (0.7%–0.8%). Similarly, pH values ranged from 5.8 (CTRL) to 5.9 (OH12.5), with no significant differences among samples (*P* > 0.05), indicating that the heat processing method did not affect product acidity.

Cooking loss ranged from 5.68 % (OH10) to 7.14 % (OH15), with CONV and OH12.5 showing intermediate values (Table 5), without significant differences observed (*P* < 0.05). This trend indicated that the different methods used resulted in comparable weight loss during processing. This similarity suggested product yield and moisture retention were not significantly affected by the heating method, an important consideration for industrial adoption. The numerical trend toward higher cooking loss at higher electric field strengths (OH15> OH12.5> OH10) might be attributed to accelerated protein denaturation and moisture expulsion, consistent with the increased firmness observed for OH‐treated samples.

WHC varied significantly among treatments (*P* < 0.05, Table 5). CTRL and OH15 showed the highest WHC (57.70% and 57.19%, respectively). CONV (54.53%) and OH10 (49.96%) showed intermediate values, while OH12.5 exhibited the lowest WHC (46.06 %, *P* < 0.05). These results indicated that higher electric field strengths (OH15) preserved WHC comparable to the raw sample (CTRL). WHC reduction in other OH samples could be attributed to longer OH processing, heat‐induced protein denaturation, and diminishing WHC of meat proteins (Li et al. 2018).

No significant difference was observed for water activity (*a*_w_, *P* > 0.05) in burger samples, with values between 0.93 (OH12.5) and 0.94 (CONV), which indicated no effect of heat processing on free water availability.

Lipid oxidation varied significantly between OH10 and other samples (*P* < 0.05, Table 5), with OH10 exhibiting the highest TBARS value (0.16 mg MDA/kg). As well, no significant differences were observed among CTRL, CONV, OH12.5, and OH15 (*P* > 0.05). This trend could be explained due to the effect of prolonged processing time (*T*_OH10_ = 82 min) and Fenton‐like reactions occurring at the titanium electrodes during ohmic heating processing (Sarkis et al. 2013).

Titanium is a reactive metal with a high thermodynamic affinity for oxygen. Upon exposure to air, its surface spontaneously and instantly forms a thin (2–7 nm), stable, passivating layer of titanium dioxide (TiO_2_) (Hou et al. 2023; Kim et al. 2022). This native oxide layer is present on the electrodes at the start of ohmic heating and serves as the reactive interface between the electrode and the food matrix. Auger electron spectroscopy and X‐ray photoelectron spectroscopy have confirmed that the first 20–50 Å of titanium exposed to air consists of mixed titanium oxides, including substoichiometric species (TiO to TiO_2_) that exhibit semiconducting properties (Cho et al. 2023; Hou et al. 2023; Kim et al. 2022).

During ohmic heating, the applied electric field induced water electrolysis at the electrode‐food interface, generating hydrogen peroxide (H_2_O_2_) in situ (Kriem et al. 2023; Zwane et al. 2021). When H_2_O_2_ contacts the TiO_2_‐coated titanium surface, a Fenton‐like reaction might occur. The Ti^4^^+^ sites on the TiO_2_ surface are reduced to Ti^3^^+^ by H_2_O_2_, and these Ti^3^^+^ species subsequently react with additional H_2_O_2_ to generate highly reactive hydroxyl radicals (•OH) (Eltaweil et al. 2025). This redox cycling (Ti^4^^+^ ⇌ Ti^3^^+^) is catalytic, meaning the titanium surface is not consumed but continuously generates reactive oxygen species as long as H_2_O_2_ is present and the electric field is maintained. This mechanism is well established in electro‐Fenton systems for water treatment, where titanium‐based electrodes activate H_2_O_2_ to produce hydroxyl radicals for pollutant degradation (Eltaweil et al. 2025; Kriem et al. 2023; Zwane et al. 2021).

Although the Fenton‐like mechanism probably occurred in all OH processing, the substantially longer processing time of OH10 allowed extended H_2_O_2_ generation, more catalytic cycles of Ti^4^^+^/Ti^3^^+^ redox, greater cumulative hydroxyl radical production, and prolonged exposure of lipids to oxidative stress (Eltaweil et al. 2025; Ferreira et al. 2021; Kriem et al. 2023; Sarkis et al. 2013). In contrast, the shorter processing time of OH15 (17 min) and OH12.5 (36 min) limited radical‐mediated oxidative damage, resulting in TBARS values comparable to CONV and CTRL.

This suggested that the primary driver of lipid oxidation in OH meat products was not the electric field strength per se, but rather the cumulative oxidative exposure time. Thus, optimizing OH parameters to minimize processing time (e.g., by using higher electric field strengths) could mitigate unwanted lipid oxidation while preserving other quality attributes. Furthermore, lipid oxidation levels were low (0.11–0.16 mg MDA/kg, *P* < 0.05) and below the threshold of 0.5 mg MDA/kg typically associated with detectable rancidity in cooked meat products (Meremäe et al. 2026). These findings indicated that both conventional grilling and ohmic heating resulted in minimal lipid oxidation when measured immediately after processing

The titanium electrodes used in this study were naturally covered with a thin, stable, passivating layer of titanium dioxide, as a spontaneously formed titanium ability (Cho et al. 2023). When an electric field (5–7.5 V/cm) was applied at 60 Hz for extended periods (17–82 min), with the electrodes in direct contact with the moist, protein‐rich burger matrix (≈61% moisture), electrochemical reactions (faradaic) could occur at the electrode‐food interface (Ferreira et al. 2021). These reactions could be facilitated by the ionic conductivity of the food's aqueous phase, which contained dissolved salts (e.g., NaCl from seasoning) and other ionic species that enable current flow through the solid matrix.

The primary faradaic reaction is water electrolysis, which generates hydrogen peroxide (H_2_O_2_) at the electrode surfaces. Additionally, the TiO_2_ surface layer can participate in redox cycling (Ti^4^^+^ ⇌ Ti^3^^+^) in the presence of H_2_O_2_, producing hydroxyl radicals (•OH) via a Fenton‐like mechanism (Eltaweil et al. 2025). However, unlike liquid foods where the electrode is fully submerged, the solid burger matrix might limit the extent of these reactions due to restricted water and ion mobility at the electrode‐food contact interface.

A recognized practical hurdle for the industrial adoption of ohmic heating in solid foods is the potential for electrode corrosion and subsequent migration of metal species into the product (Ferreira et al. 2021). In the present study, no visible corrosion, pitting, or mass loss was observed on the titanium electrodes after processing. However, it is worth investigating, and future directions intend to quantify titanium and other potential metal species in the cooked burgers by inductively coupled plasma mass spectrometry (ICP‐MS). Such analysis is essential for assessing the safety and regulatory compliance of OH‐processed meat products. Additionally, exploring higher frequency operation (kHz range) could further minimize faradaic reactions and enhance the industrial viability of ohmic heating for solid foods (Jan et al. 2023).

The color analysis revealed distinct differences between the CTRL and the heat‐processed samples. These variations reflected the impact of heating methods on surface appearance, likely influenced by biochemical reactions such as protein denaturation, Maillard browning, and pigment oxidation (Valentim et al. 2024). Thus, CIELAB parameters (L*, a*, b*), derived metrics (C*, *h°*), and total color difference (∆E*) are shown in Table 5.

Lightness (L*) values indicated that OH10 burger was significantly brighter than the raw CTRL and CONV samples (*P* < 0.05, Table 5). OH12.5 and OH15 showed intermediate values compared to the other samples (*P* > 0.05, Table 5). Increased lightness in OH samples is consistent with reported effects of volumetric heating, which minimizes surface dehydration and crust formation, resulting in a paler appearance compared to grilling, where heat transfer occurs primarily through conduction from the grill surface (Valentim et al. 2024).

Red–green chromaticity (a*) decreased significantly in OH burgers (in comparison to CTRL and CONV (*P* < 0.05, Table 5). This reduction indicates loss of redness, which is typically associated with myoglobin denaturation during heating. Myoglobin transitions from red oxymyoglobin to brown metmyoglobin as temperature increases (Valentim et al. 2024). In OH processing, rapid volumetric heating may accelerate this transition. In CONV, as the heat was conducted from the hot surface of grill plates to the burger patty, the myoglobin denatured faster and changed color from its raw pink hue to a tan or greyish–brown color, which is typical for thoroughly cooked meat. (Valentim et al. 2024); also explaining higher b* value.

Yellow‐blue chromaticity (b*) was significantly higher in CONV than CTRL and OH10 (*P* < 0.05), while OH12.5 and OH15 did not differ significantly from CTRL (*P* > 0.05, Table 4). The higher b* value in CONV likely arose from enhanced Maillard reactions on the surface due to toasting under high dry heat, producing yellow–brown melanoidins (Valentim et al. 2024). Ohmic heating, which passes electrical current directly through the food matrix, may suppress these reactions, resulting in lower yellowness, particularly at lower field strengths such as OH10. At higher voltages, increased heating rates could partially promote the Maillard reaction, explaining the slight rise in b*.

Chroma (C*), representing color saturation, followed a similar pattern to b*, with CONV showing the most vivid color, significantly higher than OH10 and OH15 (*P* < 0.05) and comparable to CTR and OH12.5 (*P* > 0.05, Table 5). This indicated that grilling may intensify overall color vibrancy through browning compounds, whereas OH at lower intensities yields duller, more muted tones (Valentim et al. 2024). Hue angle (h*) increased progressively from CTRL to CONV, and further in OH samples (*P* < 0.05), shifting toward more yellow hues, reflecting the relative dominance of b* over a* in heated samples (Valentim et al. 2024). Total color difference (∆E*) relative to CTRL was similar across all processed samples, with no significant differences (*P* < 0.05; Table 5), suggesting that both heating methods induce comparable overall visual changes despite varying mechanisms. Values above 3–5 typically indicate noticeable differences to the human eye (Sakiroff et al. 2022), confirming that heat processing visibly altered burger appearance.

### 3.4 Rheological Properties of Pork Burgers

Table 6 presents the rheological parameters of pork burgers. Uniaxial analysis revealed the following trends. The modulus of deformability (𝐸_𝐷_), which represents burger stiffness, was determined from the initial linear segment of the stress‐strain curve. 𝐸_𝐷_ differed significantly among treatments (*P* < 0.05).

**TABLE 6:** Rheological parameters of pork burger samples.

Sample	CTRL			CONV			OH10			OH12.5			OH15		
$E_{D}$ (kPa)	24.60	±	1.25^e^	33.9	±	1.43^d^	78.3	±	1.67^c^	96.1	±	1.40^a^	82.7	±	2.15^b^
$\varepsilon_{20}$ (‐)	0.88	±	0.09^a^	0.91	±	0.06^a^	0.85	±	0.14^a^	0.82	±	0.08^a^	0.90	±	0.16^a^
$\sigma_{20}$ (kPa)	7.37	±	0.41^c^	54.03	±	1.08^b^	67.29	±	1.93^ab^	73.79	±	1.25^a^	69.94	±	1.89^ab^
$W_{c}$ (kJ/m^3^)	4.72	±	0.31^c^	28.29	±	0.84^b^	34.63	±	0.88^ab^	36.63	±	0.90^a^	37.09	±	1.38^a^
$J_{0}$ × 10^−7^ (Pa^−1^)	1.33	±	0.52^b^	16.50	±	6.5^a^	16.30	±	4.88^a^	14.60	±	3.25^a^	15.71	±	0.58^a^
$J_{1}$ × 10^−5^ (Pa^−1^)	1.10	±	0.37^a^	0.873	±	0.25^a^	0.41	±	0.13^b^	0.72	±	0.02^ab^	0.30	±	0.04^b^
τ (s)	19.60	±	8.09^a^	10.60	±	1.80^b^	11.90	±	1.33^b^	10.90	±	1.82^b^	11.00	±	0.637^b^
$\eta_{N}$ × 10^6^ (Pa·s)	9.31	±	0.93^c^	37.00	±	8.33^b^	74.29	±	5.71^a^	79.90	±	4.54^a^	84.70	±	5.87^a^

*Note*: Different letters in the same row indicate statistically significant differences (*p* < 0.05, Fisher's LSD). $E_{D}$: deformability; $\varepsilon_{20}$: strain at peak stress; $\sigma_{20}$: hardness; $W_{c}$: work of compression; $J_{0}$: elastic compliance; $J_{1}$: viscoelastic compliance; *τ*: retardation time; $\eta_{N}$: Newtonian viscosity; CTRL: unprocessed raw pork burger; CONV: conventional grilled pork burger; OH10: ohmic heated at 5 V/cm pork burger; OH12.5: ohmic heated at 6.3 V/cm pork burger; OH15: ohmic heated at 7.5 V/cm pork burger.

The CTRL sample exhibited the lowest 𝐸_𝐷_ (24.6 kPa), indicating a softer texture. In contrast, OH10 significantly increased 𝐸_𝐷_ to 78.3 kPa (*P* < 0.05), demonstrating a firmer texture compared to CTRL and CONV (33.9 kPa, *P* < 0.05). The highest 𝐸_𝐷_ values were observed for OH samples (*P* < 0.05; Table 6), suggesting that OH further enhances burger firmness. Overall, OH (*P* < 0.05), particularly at higher voltages, induced a substantial increase in the modulus of deformability, likely due to more uniform heat penetration leading to protein denaturation and water loss (Lefèvre et al. 2022).

Strain at peak stress (𝜀_20_) reflected the deformation of the pork burger samples at 20% compression. All samples showed similar 𝜀_20_ values, ranging from 0.8 to 0.9, with no significant differences (*P* > 0.05; Table 6), suggesting that the deformation behavior of the burger samples was not significantly altered by the heating methods applied. Hardness (𝜎_20_), defined as the peak stress at 20% compression, was significantly lower for CTRL (7.37 kPa, *P* < 0.05) compared to all treated samples (Table 6).

The work to 20% compression (𝑊_20_) quantifies the energy required to achieve 20% compression. Higher 𝑊_20_ values indicate greater resistance to compression, implying a firmer, tougher texture. The CTRL sample required the least energy (4.72 kJ/m^3^), while the energy required for the heat‐processed samples was significantly higher (*P* < 0.05, Table 6). The increased 𝑊_20_ in ohmic‐heated samples suggested greater resistance to deformation, likely due to the increased protein cross‐linking and water loss, leading to a denser and firmer structure.

In Table 6, it can also be seen that the rheological parameters were obtained from the four‐component Burger model. These parameters offered valuable information about the viscoelastic behavior of the Burger samples during the creep test. The instantaneous elastic compliance (𝐽_0_) expresses a material's instant response to deformation. The 𝐽_0_ was significantly lower in the CTRL sample (1.33 × 10^−7^ Pa^−1^) compared to the heat‐processed samples, which showed significantly higher 𝐽_0_ values (*P* < 0.05, Table 6). It seemed that heat was responsible for increasing the immediate elastic response of the burger samples, reflecting a higher initial compliance and suggesting the internal structure was more flexible at the onset of stress application (Gao et al. 2025).

The viscoelastic compliance parameter (𝐽_1_) indicates how the material gradually deforms under sustained stress, with higher values signifying more pronounced viscoelastic behavior, where deformation persists after the initial elastic response. The 𝐽_1_ values were also affected by OH. The CTRL sample showed a higher 𝐽_1_ (1.10 × 10^−5^ Pa^−1^) compared to OH10 (0.41 × 10^−5^ Pa^−1^) and OH15 (0.30 × 10^−5^ Pa^−1^), indicating a more pronounced viscoelastic behavior in the non‐heated sample. OH12.5 (0.72 × 10^−5^ Pa^−1^) did not show significant differences from CTRL (*P* > 0.5), suggesting that while some OH treatments reduced viscoelastic compliance, others did not.

The retardation time (*τ*) reflects the time‐dependent response of the samples. The CTRL sample exhibited the longest τ (19.60 s), significantly different from heat‐processed samples (*P* < 0.05). Heat‐processed samples showed reduced retardation times, indicating a quicker stress relaxation behavior, suggesting that heating accelerated the stress relaxation process, likely due to structural changes at the molecular level.

The Newtonian viscosity (𝜂_𝑁_) was higher in the ohmic‐heated samples (*P* < 0.05, Table 6), compared to CTRL (9.31 × 10^6^ Pa·s) and CONV (37.00 × 10^6^ Pa·s) (*P* < 0.05). OH significantly increased the resistance to flow, suggesting a more viscous nature of the burger matrix post‐treatment.

The rheological analysis demonstrated that OH significantly enhanced the textural and mechanical properties of burger samples when compared to untreated (CTRL) and grilled (CONV) ones. Notably, OH, particularly at higher voltages, resulted in markedly firmer and tougher burgers, as evidenced by the increased modulus of deformability and higher hardness (*σ*_20_). These improvements in firmness and toughness render the burgers more resistant to deformation and more durable during handling and cooking processes. Furthermore, the enhanced elastic compliance and consistent fracture strain suggested OH burgers maintained a balanced texture, combining both firmness and flexibility. The quicker stress relaxation (lower *τ*) and higher resistance to flow (higher 𝜂_𝑁_) further indicate enhanced structural integrity of the burgers.

Souppez et al. (2025) observed a significant difference in the cooked yield modulus and flexural yield strain between beef and plant‐based burgers (*P* < 0.05), suggesting that cooking methods indeed influenced structural changes in the burger matrix. For instance, baking, as opposed to grilling, resulted in higher water retention in beef burgers, leading to smaller variations in mechanical properties between raw and cooked states (Souppez et al. 2025). This finding aligns with the present pork burger data, where OH rapid and uniform heat distribution enhanced firmness and hardness, likely due to similar mechanisms of protein cross‐linking and moisture reduction.

Souppez et al. (2025) also reported significant differences in yield strain for both raw and cooked states (*P* < 0.05), particularly influenced by water content. This discrepancy may be attributed to the compositional differences between pork and beef or plant‐based burgers, where water retention plays a more pronounced role in beef and plant‐based formulations (Souppez et al. 2025). This observation suggested that water acts as a plasticizer, enhancing extensibility in beef burgers but not significantly in pork burgers under OH.

The viscoelastic behavior evaluated in OH10 and OH15 samples indicated a more elastic and viscous structure post‐OH, likely due to protein denaturation and a denser matrix. This could be supported by Souppez et al. (2025), who similarly noted that protein content in beef burgers contributed to a more pliable texture, particularly in the cooked state, where yield strain differences were less pronounced. This fact suggested that protein type and content significantly influence viscoelastic behavior, with pork burgers showing enhanced elastic response due to OH, while beef and plant‐based burgers exhibit variations driven by protein melting characteristics and cooking methods. The increased 𝑊_20_ in OH samples indicated a firmer texture, corroborating Souppez et al. (2025), who found hardness, cohesiveness, and chewiness as distinguishing factors between meat‐based and plant‐based burgers. The increased firmness in OH pork burgers aligned with the higher yield modulus and chewiness in cooked beef burgers, suggesting that OH, as baking, enhanced structural integrity through similar mechanisms of protein cross‐linking and moisture loss.

Therefore, OH modified the rheological features of samples, resulting in firmer, tougher, and more viscous burgers with improved structural integrity (*P* < 0.05). These findings indicated that OH proved to be an effective method for altering the textural attributes and mechanical stability of burger products.

### 3.5 Volatile Organic Compounds Profile for Burgers Aromas Evaluation

Various VOCs were detected using the electronic nose analysis, from which 26 of them were considered important for the samples under study (relative standard deviation below 50% and sensor intensity considered). Table 7 lists these compounds and their corresponding sources contributing to the burger's aroma profile.

**TABLE 7:** The most relevant volatile organic compounds (VOCs) identified in pork burger samples (relative standard deviations < 30%). Compounds are grouped by chemical classification and categorized based on their primary sources of origin. The VOCs were identified in all burger samples.

Chemical groups	Compound	Source	Reference
Alcohols	1‐Octanol	Meat's lipid oxidation	
Aldehydes	Butanal		Wang et al. (2023)
	Propanal		
	2‐Methyl propanal	Maillard reaction	
	3‐Methyl butanal		
Alkanes	Butane	Meat heating	Lee et al. (2020)
	Heptane		
Carboxylic acids	Propanoic acid	Meat's lipid oxidation	Mu et al. (2023)
Esters	Benzyl acetate	Seasonings	Walasek‐Janusz et al. (2024)
	Ethyl butyrate	Meat's lipid oxidation or esterification reaction between alcohols and fatty acids during cooking	Sohail et al. (2022)
	Ethyl hexanoate	Meat's lipid oxidation or esterification reaction between alcohols and fatty acids during cooking	
	Ethyl octanoate	Meat's lipid oxidation	
	Hexyl acetate	Meat's lipid oxidation or esterification reaction between alcohols and fatty acids during cooking	
	Isoamyl acetate	Meat cooking and esterification	
Lactones	4‐Octanolide	Meat's lipid oxidation or Maillard reaction	Ahamed et al. (2024)
Miscellaneous	Maltol	Maillard reaction and garlic	
Phenolic compounds	Thymol	Oregano and cumin	Soncu et al. (2020)
	Anethole	Nutmeg and cumin	Walasek‐Janusz et al. (2024)
	Vanilin	Nutmeg	
Pyrazines	2,6‐Dimethylpyrazine	Maillard reaction	Ahamed et al. (2024)
Sulfur compounds	Dimethyl sulfide	Garlic and black pepper	Sohail et al. (2022)
	Dimethyl trisulfide		Walasek‐Janusz et al. (2024)
Terpenes	α‐Terpinene	Black pepper and oregano	
	Myrcene		
	L‐Limonene	Black pepper and nutmeg	
	β‐Pinene		

Meat‐derived compounds primarily included aldehydes (e.g., propanal, butanal), alcohols (e.g., 1‐octanol), alkanes (e.g., butane), and esters (e.g., ethyl hexanoate), which are often formed through lipid oxidation, Maillard reactions, or protein degradation (Ahamed et al. 2024). Seasonings (garlic, black pepper, nutmeg, cumin, oregano) contributed aromatic terpenes (e.g., β‐pinene, thymol) and sulfur compounds (e.g., dimethyl sulfide). Oregano‐derived terpenes, such as β‐pinene and myrcene, added fresh, herbal notes. β‐pinene provided woody and pine‐like aromas, while myrcene contributed earthy nuances (Walasek‐Janusz et al. 2024). Thymol, found in both oregano and cumin, imparted a pungent, slightly medicinal scent (Soncu et al. 2020). Some compounds, such as 2,6‐dimethylpyrazine and maltol, resulted from cooking interactions between meat and seasonings, contributing toasted, nutty, and caramel‐like aromas, respectively (Sun et al. 2022). Fruity esters (e.g., ethyl butyrate, isoamyl acetate) helped balance sulfurous notes (e.g., dimethyl sulfide), which developed during cooking and imparted earthy, slightly sweet, cabbage‐like aromas, enhancing the umami character (Sohail et al. 2022).

While the Maillard reaction is a key pathway for aroma formation in heated meat products (Santos et al. 2024a), thermal processing also generates additional compounds due to heat exposure (Bleicher et al. 2022). Moderate lipid oxidation enhanced desirable meat flavors, producing volatile substances such as hexanal and benzaldehyde depending on cooking temperature and time (Wang et al. 2023). In this study, propanoic acid, ethyl butyrate, ethyl hexanoate, ethyl octanoate, hexyl acetate, isoamyl acetate, and 4‐octanolide were identified as products of lipid oxidation during cooking. Aldehydes such as propanal contributed fresh, fruity, apple‐like notes, while 3‐methylbutanal imparted malty, nutty qualities associated with roasted flavors (Mu et al. 2023).

DFA is a supervised statistical method that transforms original variables into new discriminant functions to maximize separation between predefined groups (Zhao et al. 2024). Thus, DFA was performed to differentiate aroma profiles among sample groups. The first two discriminant functions were significant (*P* < 0.05), accounting for 95.76% (DF1) and 4.24% (DF2) of the total variance (cumulative 100%). Additional discriminant functions (DF3–DF5) were not significant (*P* > 0.05) and were not reported.

**FIGURE 3:**
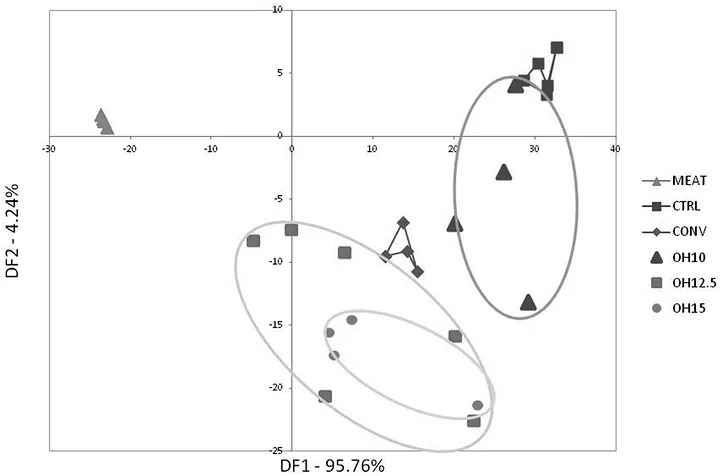
Discriminant analysis (DA) of burger samples analyzed in six replicates, and grouped based on aroma similarities. Additional functions ((DF3, DF4, DF5) were not significant (*P* > 0.05, Wilks' lambda), therefore not shown. MEAT: standard raw pork meat; CTRL: unprocessed raw pork burger; CONV: conventional grilled pork burger; OH10: ohmic heated at 5 V/cm pork burger; OH12.5: ohmic heated at 6.3 V/cm pork burger; OH15: ohmic heated at 7.5 V/cm pork burger. The seasoning mix standard is not shown in the plot to improve the clarity of sample differentiation.

The DFA plot (Figure 3) revealed two main findings. First, raw samples (MEAT and CTRL) were clearly separated from all heat‐processed samples (CONV, OH10, OH12.5, OH15), indicating that cooking induces substantial changes in the VOC profile regardless of the heating method. Second, among heat‐processed samples, CONV and all OH treatments clustered together with no discernible separation, confirming that no significant differences in volatile organic compounds were detected between conventional grilling and ohmic heating under the conditions tested. The first discriminant function (DF1, 95.76%) primarily separated raw from cooked samples, while the second discriminant function (DF2, 4.24%) did not separate CONV from OH treatments, consistent with the absence of significant differences between heating methods.

It is important to note that the VOC extraction method itself relies on heating (80°C for 15 min). Consequently, the CTRL sample was also cooked during analysis, generating similar volatile compounds as the heat‐processed samples. This explains why CTRL and heat‐processed samples show some overlap in the DFA plot. Notably, OH10 samples showed slight similarity to CTRL, likely because the long processing time (90 min) and lower electric field strength (5 V/cm) of OH10 resulted in milder heating compared to OH12.5 and OH15, making its VOC profile more comparable to the heated CTRL sample.

The aroma profile of pork burgers was shaped by the integration of Maillard reaction products, seasoning‐derived compounds, and additional volatile organic compounds generated during thermal processing. The combination of savory, herbal, sweet, and fruity notes resulted in a well‐balanced and multidimensional aroma profile.

### 3.6 Biological Functionality of Pork Burgers

**FIGURE 4:**
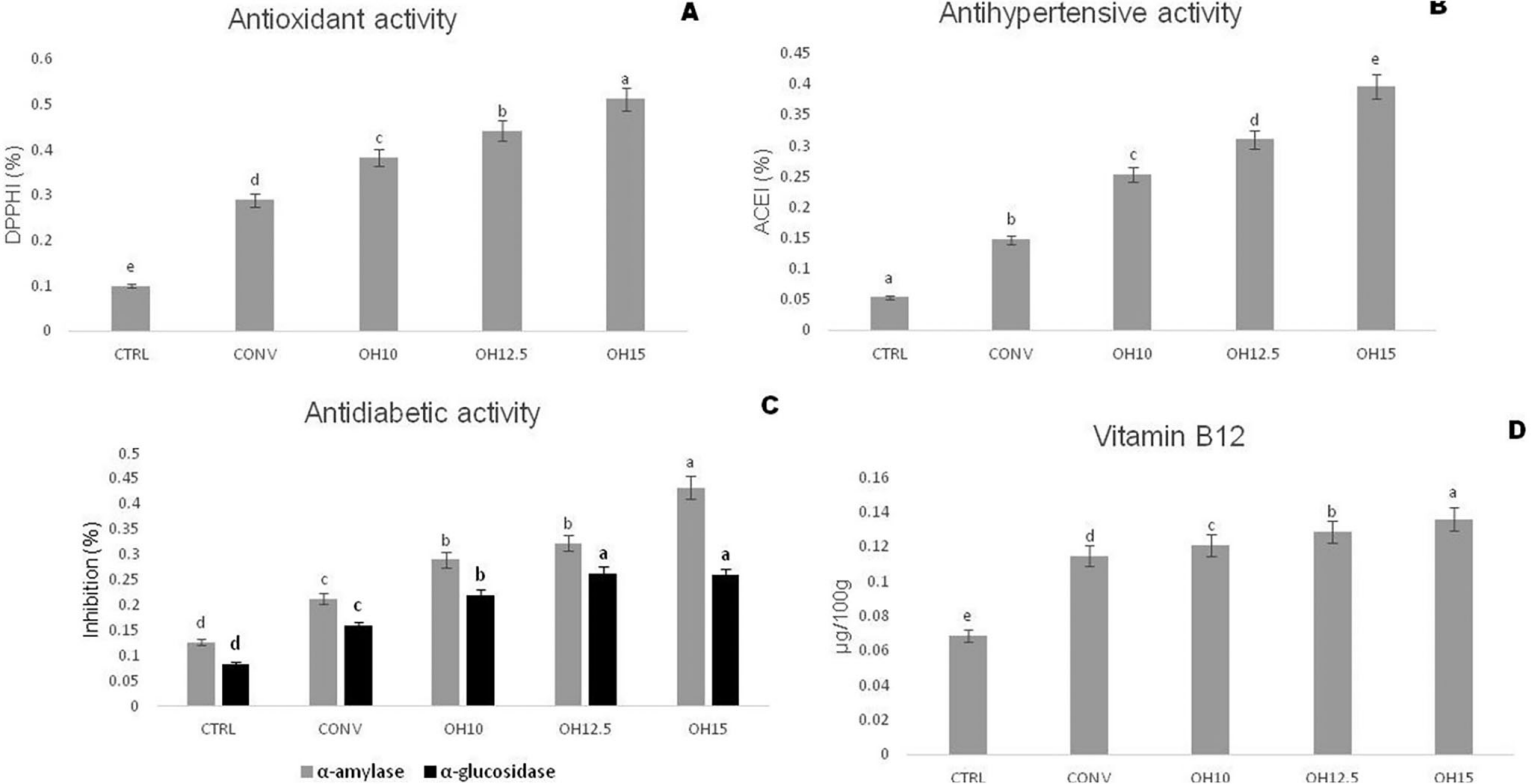
(A) Antioxidant (2,2‐diphenyl‐1‐picrylhydrazyl radical—DPPH inhibition), (B) antihypertensive (angiotensin‐converting enzyme—ACE inhibition) and (C) antidiabetic (α‐amylase and α‐glucosidase inhibition) activities, and (D) vitamin B12 content in pork burgers processed by conventional grilling or ohmic heating. CTRL: unprocessed raw pork burger; CONV: conventional grilled pork burger; OH10: ohmic heated at 5 V/cm pork burger; OH12.5: ohmic heated at 6.3 V/cm pork burger; OH15: ohmic heated at 7.5 V/cm pork burger.

The in vitro bioactivity and vitamin B12 content of pork burgers varied significantly across heat processing methods (*P* < 0.05, Figure 4). The CTRL exhibited the lowest measured activities, with DPPH radical scavenging activity at 0.100% (Figure 4A), ACE inhibitory (ACEI) activity at 0.053% (Figure 4B), and antidiabetic‐related enzyme inhibition (Figure 4C) at 0.127% (α‐amylase inhibition) and 0.083% (α‐glucosidase inhibition), alongside a vitamin B12 content of 0.07 ± 0.01 µg/100 g (*P* < 0.05). CONV showed moderate improvements, with DPPH at 0.29%, ACEI at 0.15%, α‐amylase inhibition at 0.21%, α‐glucosidase inhibition at 0.16%, and vitamin B12 content of 0.11 ± 0.01 µg/100 g (*P* < 0.05). OH samples demonstrated superior in vitro activities, with OH10 achieving DPPH at 0.38%, ACEI at 0.25%, α‐amylase inhibition at 0.29%, and α‐glucosidase inhibition at 0.22%; OH12.5 at 0.44%, 0.31%, 0.32%, and 0.26%; and OH15 at 0.51%, 0.40%, 0.43%, and 0.26%, respectively (*P* < 0.05). Vitamin B12 content also increased with ohmic heating (*P* < 0.05), reaching 0.12 ± 0.01 µg/100 g (OH10), 0.13 ± 0.01 µg/100 g (OH12.5), and 0.14 ± 0.01 µg/100 g (OH15). These results suggested that OH volumetric heat generation and electroporation enhanced the retention and extraction of bioactive compounds and preserved heat‐sensitive nutrients compared to conventional grilling.

The improvement in CONV bioactive compounds over CTRL could be explained by a concentration effect (moisture loss), mild temperature heat processing (75°C), and Maillard reaction product formation during heating, which may contribute to the release of bioactive peptides through mild heating (Bolchini et al. 2025). Mild heating also enhances the in vitro bioactivity of seasonings (Leong and Chang 2024), which could contribute to a baseline level due to the low pork burger seasoning content.

Luasiri et al. (2024) verified that cooking goat meat at approximately 70°C for 30 min, similar to conditions used in this study, enhanced the release of bioactive peptides in the loin cut, exhibiting 5% to 10% higher ACE and DPPH inhibitions, respectively. At milder heating (< 90°C), the α‐helix protein structure remains intact, preserving hydrated protein networks and minimizing oxidative damage (Katemala et al. 2023). Besides the heating improvement, OH heat processing also exhibited an electrical feature, the electroporation phenomenon, which, in synergy with uniform homogeneous heating, probably explains the superior in vitro bioactivity of OH samples. Electroporation disrupts cellular structures, extracting intracellular compounds into the burger matrix (Yu et al. 2024). Thus, preserving bioactive peptides in beef patties and pork emulsions (González‐Osuna et al. 2024). As well. Zhang et al. (2025) used radio frequency heating combined with complementary technologies like ultraviolet, vacuum drying, or cold shock to improve the retention of bioactive compounds such as polyphenols, flavonoids, and antioxidants in processed foods. Optimal temperatures to minimize thermal degradation were between 70°C and 85°C, with rapid heating and reduced oxygen exposure playing key roles in bioactive preservation (Zhang et al. 2025).

Regarding vitamin B12 (Figure 4D), cobalamin is thermally stable at temperatures below 100°C and cannot be synthesized during heating (Moravcová et al. 2025). Therefore, the higher concentrations observed in heat‐processed samples (CONV and OH) compared to raw CTRL (0.07 µg/100 g) reflect improved extractability rather than retention of a labile nutrient. Heat‐induced protein denaturation likely releases cobalamin from the protein matrix, making it more accessible to the extraction solvents used in the HPLC method (Czerwonka et al. 2014). As well, since all samples were subjected to the same extraction procedure, the comparative differences among treatments remain valid.

Among processed samples, vitamin B12 content increased with OH in a voltage‐dependent manner: 0.12 µg/100 g (OH10), 0.13 µg/100 g (OH12.5) and 0.14 µg/100 g (OH15), compared to 0.11 µg/100 g for CONV (*P* < 0.05). This pattern suggests that OH processing, particularly at higher electric field strengths (OH15), further enhances cobalamin extractability, possibly due to more extensive protein denaturation or electroporation‐induced cell disruption (Hu et al. 2026; Santos et al. 2024; Yu et al. 2024).

The synergistic effects of uniform volumetric heating and electroporation likely enhanced the release and preservation of bioactive peptides and heat‐sensitive nutrients, outperforming CONV's surface‐focused heating, which may lead to greater nutrient losses due to protein denaturation and leaching. These findings align with Müller et al. (2020), who noted OH's ability to improve food quality by minimizing thermal degradation of bioactive compounds. OH provided higher extraction of total phenolic compounds from winemaking by‐products (48% more) compared to conventional processing (Coelho et al. 2023), confirming this technology's quality preservation mechanism. Therefore, optimizing OH parameters, such as electric field strength and processing time, could further enhance in vitro bioactivity and nutrient retention, offering a promising approach for producing functional meat products.

In the end, OH demonstrated potential in increasing biological activities by inhibiting DPPH and ACE. However, these activities were extremely low, and it could not be stated that burgers processed with OH are a source of antioxidant, antihypertensive, and antidiabetic activity.

## 4 Conclusions

This study demonstrates that ohmic heating (OH) offers advantages for pork burger processing compared to conventional grilling (CONV), particularly in energy efficiency (40% reduction in specific energy consumption) and microbial inactivation (TAMB ≈ 95% reduction). OH also produced firmer, tougher burgers. However, these benefits came at the cost of substantially longer processing times (OH: 17–82 min) in comparison to CONV (7 min). Therefore, for industrial applications where throughput is critical, these trade‐offs must be carefully considered. Higher electric field strengths (e.g., OH15) should therefore be prioritized to balance energy savings with acceptable processing times.

Acknowledging the limitations of the present study, a mathematical projection of OH at equivalent CONV electrical power suggested potential for industrial scale‐up. A realistic scenario for industrial equipment yielded a projected processing time of 3 min, 57% faster processing time than CONV, delivering the same total energy (0.03 kWh). This projection reinforces that OH operated at higher power levels could overcome the processing time limitations observed in this laboratory‐scale study.

Nevertheless, claims of global sustainability benefits would require supporting life cycle assessment (LCA) data, which was beyond the present scope. Future pilot‐scale studies should evaluate OH under continuous production conditions, assessing consumer acceptance through sensory analysis, and incorporate LCA to quantify the true environmental footprint before positioning OH as a broadly sustainable alternative to conventional grilling.

## Author Contributions

**Celso F. Balthazar**: investigation, funding acquisition, writing – original draft, methodology, validation, visualization, formal analysis, project administration, resources, supervision, data curation, conceptualization, software, writing – review and editing. **Jonas T. Guimaraes**: methodology, writing – review and editing, visualization, software, formal analysis, validation. **Rodrigo N. Cavalcanti**: methodology, software, formal analysis, validation, visualization, writing – original draft. **Andrea G. Mascarenhas**: methodology, formal analysis, data curation. **Thais R. C. Pereira**: methodology, formal analysis, data curation. **Renata S. L. Raices**: methodology, validation, visualization, formal analysis, supervision. **Monica Q. Freitas**: writing – review and editing, resources, supervision. **Erick A. Esmerino**: visualization, writing – review and editing, resources, supervision. **Adriano G. Cruz**: supervision, resources, visualization, writing – review and editing. **Sergio B. Mano**: visualization, writing – review and editing, supervision, resources. **Eliane T. Mársico**: visualization, writing – review and editing, resources, supervision, funding acquisition.

## Funding

Celso F. Balthazar thanks the Rio de Janeiro Research Foundation (FAPERJ) for the SEI‐260003/000156/2024 grant (E‐26/200.230/2024 and E‐26/200.231/2024). Eliane T. Mársico thanks FAPERJ for the resource provided (E‐26/201.048/2022). Andrea G. Mascarenhas thanks to Coordenação de Aperfeiçoamento de Pessoal de Nível Superior ‐ Brasil (CAPES) for the doctoral scholarship (88887.854401/2023‐00).

## Conflicts of Interest

The authors declare no conflicts of interest.
